# Sharing and organizing research products as R packages

**DOI:** 10.3758/s13428-020-01436-x

**Published:** 2020-09-01

**Authors:** Matti Vuorre, Matthew J. C. Crump

**Affiliations:** 1 Oxford Internet Institute, University of Oxford, 1 St Giles, Oxford, OX1 3JS UK; 2 Department of Psychology, Brooklyn College of CUNY, Brooklyn, NY 11210 USA

**Keywords:** Reproducibility, Research methods, R, Open data, Open science

## Abstract

A consensus on the importance of open data and reproducible code is emerging. How should data and code be shared to maximize the key desiderata of reproducibility, permanence, and accessibility? Research assets should be stored persistently in formats that are not software restrictive, and documented so that others can reproduce and extend the required computations. The sharing method should be easy to adopt by already busy researchers. We suggest the R package standard as a solution for creating, curating, and communicating research assets. The R package standard, with extensions discussed herein, provides a format for assets and metadata that satisfies the above desiderata, facilitates reproducibility, open access, and sharing of materials through online platforms like GitHub and Open Science Framework. We discuss a stack of R resources that help users create reproducible collections of research assets, from experiments to manuscripts, in the RStudio interface. We created an R package, *vertical*, to help researchers incorporate these tools into their workflows, and discuss its functionality at length in an online supplement. Together, these tools may increase the reproducibility and openness of psychological science.

## Introduction

Research projects produce experiments, data, analyses, manuscripts, posters, slides, stimuli and materials, computational models, and more. However, the potential added value of these products is not fully realized due to limited sharing and curating practices. Although more transparent communication of these research products has recently been encouraged (Houtkoop et al., 2018; Klein et al., 2018; Lindsay, 2017; Martone et al., 2018; Rouder, 2016; Rouder et al., 2019; Vanpaemel et al., 2015; Wicherts et al., 2006), these efforts often focus narrowly on sharing data (and sometimes analysis code). Further, the practical value of sharing is often limited by poor documentation, incompatible file formats, and lack of organization, resulting in low rates of reproducibility^1^ (Hardwicke et al., 2018). Standardization of protocols for sharing would be beneficial, but such standards have not emerged, possibly due to the variance of research in psychology. Instead of developing another standard, we suggest borrowing existing standards and practices from software engineering. Specifically, the R package standard, with additional R authoring tools, provides a robust framework for organizing and sharing reproducible research products.

Some advances in data-sharing standards have emerged: It is becoming more popular to share data on the Open Science Framework (OSF). However, those materials often contain idiosyncratic file organization and minimal or missing documentation for raw data. In specific areas, organization and documentation standards have emerged, (e.g., the BIDS framework in neuroscience, Gorgolewski et al., (2016), but they usually only consider data and code instead of the project as a whole. More comprehensive proposals are described in the Transparency and Openness Promotion (Nosek et al., 2015), and Peer Reviewers’ Openness initiative guidelines (Morey et al., 2016), but these fall short of describing detailed standards for organization and metadata. Additionally, these standards (if they exist) may not be widely known, recognized, agreed upon, and/or adopted at large.

We sought a standard for organizing and sharing that would adhere to the FAIR (Findable, Accessible, Interoperable, Reusable) guidelines to maximize the reuse potential of data and support “discovery through good data management” (Wilkinson et al., 2016), see also general discussion). Additionally, we recognized the added value of including other research outputs (“products”; e.g., manuscripts) beyond datasets in a reproducible collection of materials. We identified the R package standard with modern online-based workflows as a solution that does not present overwhelming overhead for already-busy researchers. Here, we discuss the R package standard for creating reproducible research material containers for data, analysis code, and metadata. Then, we introduce additional R packages for incorporating other research products into the reproducible container. These additional tools are easily accessible through our *vertical* package, thus named because an entire research project can be completed from top to bottom as a stack of R processes from within the RStudio software platform.

## R Packages

R is a programming language for statistical analyses (R Core Team, 2020); https://www.r-project.org), and RStudio is its associated integrated development environment (IDE; RStudio Team (2016)); https://www.rstudio.com/). Both are free, open source, and work on Windows, Mac, and Linux systems. These tools are already widely used among psychology researchers: In one crowd-sourced analysis, 16 of 29 analysis teams used R (Silberzahn et al., 2018), and many psychologists have already developed R packages (see also Yee and Debbie (2017), and http://r4stats.com/articles/popularity/).

A cornerstone of R is user-created packages, which contain functions, (meta)data, and documentation in a standard format that allows seamless sharing across users and operating systems. Data and functions in R packages are immediately available to others through the R console, and documentation can be viewed in R or online. We first outline how data and functions are included in R packages, and then turn to including other assets, such as manuscripts and posters. A complete guide to R packages is outside our scope (see Wickham (2015)).

**Fig. 1:**
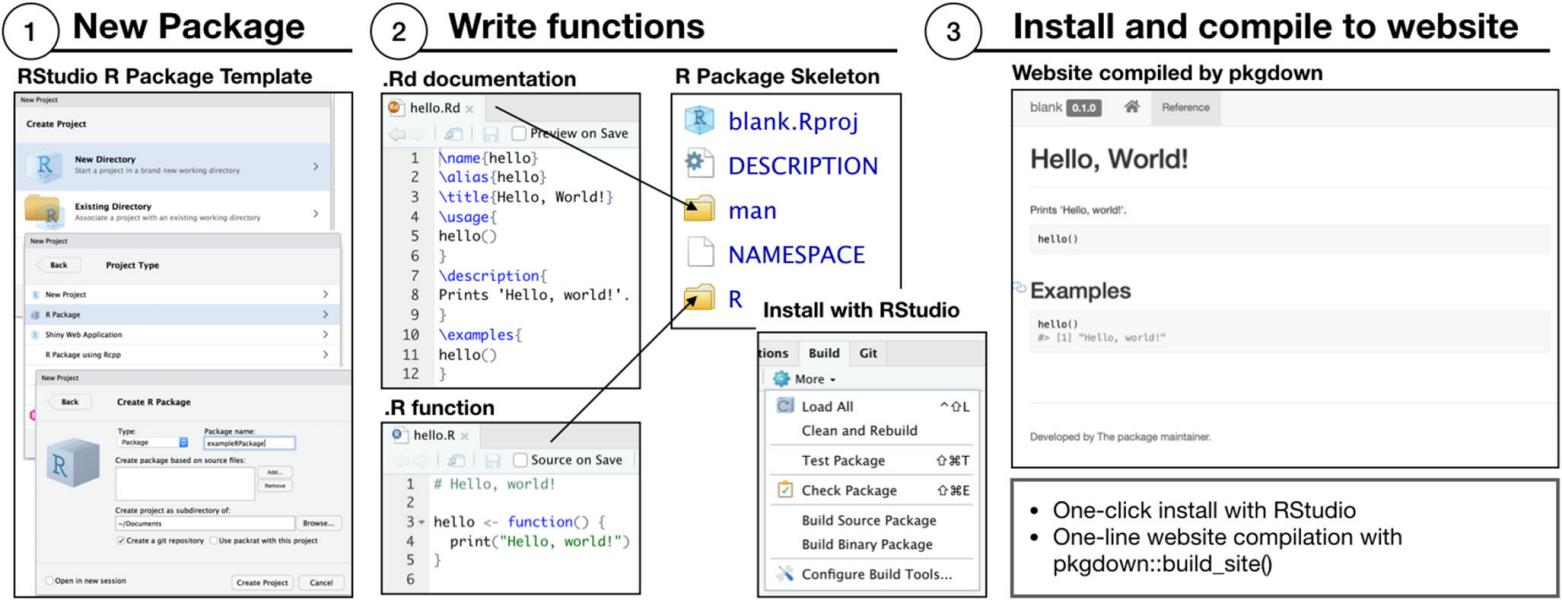
Creating an R package using the RStudio graphical user interface

When an R package is created as shown in Fig. 1, the required files and directories are automatically created. At this point, the package can be installed (click “Install and Restart” in RStudio’s “Build” tab) and shared online (see below). However, to make it useful, content needs to be added, beginning with the package’s description. That information (e.g., authors, dependencies) is written in the “DESCRIPTION” file in a machine-readable format. To edit that file, click on its name in RStudio’s File browser. The “blank.Rproj”, “man” and “NAMESPACE” files/directories shown in Fig. 1 should not be edited by users, and we thus turn to R functions.

### Functions

Researchers often develop and use custom functions in their analysis. Although it is possible to declare them directly in the analysis scripts, we suggest declaring functions using the R package standard, by placing the function’s source code into a file in the “R” directory. That directory is automatically created (see Fig. 1). Functions in R packages are portable, such that others can install the package from their R console, load it, and start using the functions immediately. Packages can also depend on other packages (and be depended on), such that R automatically installs any requirements for your functions to work appropriately. Functions within R packages are documented in a standardized manner, and the documentation for a function can be viewed in R (e.g., try ?mean) or online.

Wrapping functions into R packages also provides a reuse benefit: Functions can be difficult to find in old scripts, but easy to find and load if they are called from an existing package. Thus, formally including one’s functions in R packages facilitates reproducibility and sharing.

### Data

Broadly, there are three steps to including data in an R package: 1. placing raw data in the “data-raw” directory, 2. creating an R script that processes the raw data and creates an R data object into the “data” directory, and 3. documenting the final data object.

First, raw data in any format is stored in the “data-raw” directory to ensure that the method of sharing remains software-agnostic. If possible, that raw data should be in a text-based format, rather than proprietary formats such as SPSS files. Second, instructions for converting and cleaning the raw data into analyzable format should also be included in this directory (the raw data files should not themselves be modified). These pre-processing steps should be saved so that the complete path from raw data to results is transparent and reproducible, preferably in an R script in the “data-raw” directory. That script should end with creating an R data object in the “data” directory. Placing the R data object there ensures that it is included in the resulting R package, which makes using the data effortless for any R user, and eschews the need to download additional files. Loading the R package makes the included data immediately available in the R console.

Finally, the resulting data object in “data” should be documented in a standardized format by placing a data.R file in the “R” directory, a process discussed in more detail in our complete online supplementary tutorial (https://crumplab.github.io/vertical/), and in Wickham (2015). That standard format of documentation is especially useful because it allows datasets’ documentation to be viewed in R (e.g., type ?attitude in the R console) and online (see below).

R packages thus provide a useful standard for functions and (meta)data. However, as we will see, the package structure can be extended to include analyses, manuscripts, posters, and presentations as well. The complete project is then easily shared online, and even showcased as a website, as we will show below. To enable these additional features, many R packages have been developed (e.g., writing manuscripts with R). To facilitate their use, we have created an R package, *vertical*, that, when installed, also installs these other packages, and makes it easier to create projects that use them.

## Reproducible research projects with *vertical*

**Fig. 2:**
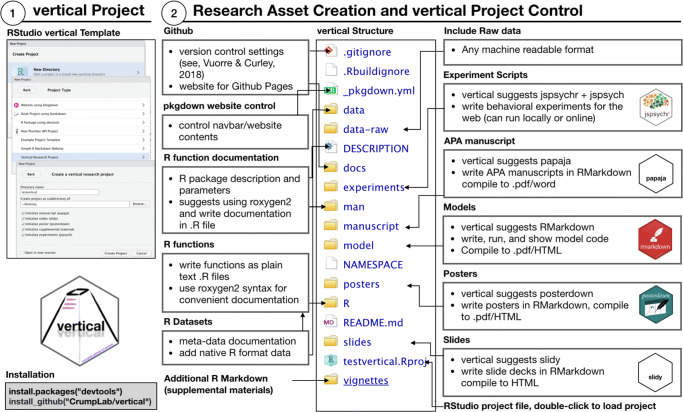
An overview of the vertical workflow. The project template suggests R Markdown modules for asset creation (manuscripts, slides, posters), that are compiled to multiple formats. Content is curated follow R package standards, then compiled and communicated in the form of an online repository (GitHub/OSF), project website, and R package

*vertical* extends the R package template with a set of additional files and directories for products such as manuscripts and posters. These assets are organized such that they can be easily shared online on GitHub or OSF, installed to R as a package, and showcased online as a website. Figure 2 illustrates the workflow of creating a *vertical* research project.

Additional benefits of packaging everything together in this manner are that the computations supporting manuscripts, supplementary analyses, data preprocessing, etc. are reproducible and use the same data in the same computational environment. Because sharing of materials is built-in, no additional work is needed after-the-fact in organizing the materials for sharing. We begin with an overview of this process, but a more exhaustive step-by-step tutorial is provided online (Crump and Vuorre, 2020).^2^ Before using *vertical*, or any packages contained therein, users must first install it in R (the *devtools* (Wickham et al., 2020) package is necessary for installing R packages from GitHub): 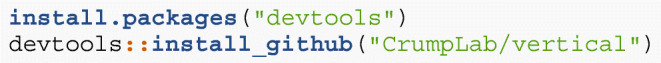


Users should then restart RStudio to make the features available. A new *vertical* research project is created through RStudio (Fig. 3.1). Figure 3.2 shows the resulting file and directory structure, which is an extension of the R package structure shown in Fig. 1. Users may enable or disable components as needed, either by (un)checking boxes (Fig. 3.1) or adding/deleting files/directories. Next, we describe the components, and then how to share the resulting research projects and showcase them as websites.

**Fig. 3:**
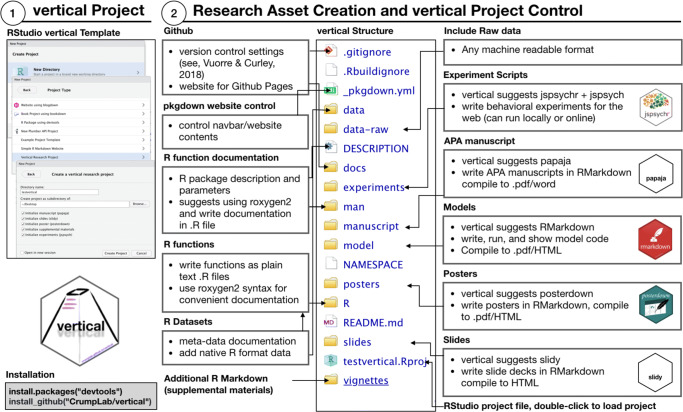
Creating a *vertical* project in RStudio (1), and description of the file structure of a *vertical* project (2)

In addition to data and functions, almost any kind of content can be included within the project. Most importantly, to create reproducible assets, such as data analyses or manuscripts, these materials should be created with R Markdown, a simple language that allows mixing prose with code (R, Python, etc.), and thus enables including one’s text and analyses in a single document. Because of its importance, we describe R Markdown first.

### Reproducible documents with R Markdown

R Markdown allows executable code snippets to be embedded alongside regular text, and simple markup for formatting code (e.g., headers using # s; Xie et al., (2018)). R Markdown documents are transparent and reproducible. In principle, recipients can see the analysis scripts, and reproduce them by compiling the document on their own machine. R Markdown documents separate content and style, and can be compiled to multiple output formats, such as PDF, HTML, and Word. Importantly, R Markdown is plain text, and thus effortless to learn. Nevertheless, a tutorial on R Markdown is beyond our scope (see Xie et al., (2018)).^3^

### Data analysis

Data analysis can be made fully transparent and reproducible with R Markdown. Above, we suggested placing any preprocessing steps in a separate file in the “data-raw” directory, and creating a documented R data object in “data”. If the data object is called mydata and the package is installed and loaded, calling mydata in R will call the data object, ready to be used in analyses. Our favored approach is to write any analyses relevant to a product (e.g., manuscript or a poster) directly into the product’s R Markdown source file. Typically, we would include data analysis code in the R Markdown source of the APA style manuscript (see below).

Nevertheless, sometimes supplementary analyses are conducted that are not included in any other document. These are most conveniently placed in R Markdown files into the “vignettes” directory. Then, when the project is compiled into a website (see below), those analyses are viewable as part of the resulting website. When a *vertical* project is created, the “vignettes” directory is automatically created with an example data analysis R Markdown script.

### APA manuscripts

Reproducible APA formatted manuscripts can be created with the *papaja* R package, that provides an R Markdown template file, and additional helper functions for creating tables and figures (Aust and Barth, 2020). For example, the R Markdown file can contain the text and code to generate the manuscript and all results. *papaja* provides several functions for reporting results: For example, *papaja*’s apa_print(model) embeds model’s statistical results directly into the manuscript, obviating the need to copy them by hand and removing human error in reporting. References can be automatically added from Zotero using the citr plugin (Aust, 2019). We refer readers to the *papaja* documentation website for more information.^4^

When a new *vertical* project is created, a *papaja* manuscript template is automatically included in the “manuscript” directory. Then, once the project is compiled, the manuscript PDF is copied to the “docs” folder, from where it can be viewed as part of the resulting website (see below).

### Posters

*vertical* includes a posters folder where poster documents can be deposited and shared. *vertical* suggests using the *posterdown* package (Thorne, 2019) for poster creation through an R Markdown document. When the provided template is used, the resulting reproducible document is viewable as part of the website.

### Slides

*vertical* includes a slides directory for slide decks in any format, which can then be included for downloading or viewing on the resulting website. There are several R Markdown options for creating reproducible slide decks in various formats. When a new *vertical* project is created, a *slidy* template is included to create web-ready presentations.^5^ Slidy presentations are viewable in a browser like typical PowerPoint or Keynote presentations, but have additional features like scrollable slides, interactive figures, and animations.

### Other materials

Other research assets may include supplementary materials, stimulus sets, code for a computational model, additional analyses, or other documents, such as blogs. Additional assets can be included by creating files in appropriate directories, and linking them to the resulting website (see below). For example, arbitrary R Markdown documents can be included in the vignettes directory. When the project is compiled (see below), R Markdown files in that directory are automatically compiled and served on the website (see Wickham and Hesselberth (2020)).

### Experiments

Finally, experiments should be placed in the experiments directory. They could be packaged as a compressed .zip file, which would then be accessible from an online repository. *vertical* suggests using *jsPsych* (De Leeuw, 2015), a JavaScript library for building browser-based experiments that can be served to the web or run locally. Selecting the *jsPsych* module during project initialization downloads the most recent version of *jsPsych* to the experiments folder. A basic HTML template is also provided, which loads the jsPsych library and presents some basic word stimuli. To further edit the experiment, users can simply edit the HTML file as they would when creating any other jsPsych experiment. When the *vertical* project is compiled, the experiment is available on the project website.

For working entirely with R Markdown, we suggest *jsPsychr*, an R package that helps creating jsPsych experiments with R Markdown in RStudio (Crump, 2019).^6^ RStudio enables passing objects between R and JavaScript, making this an attractive option for experienced R users. The template creates a sample *jsPsych* experiment produced by *jsPsychr*, which contains the R Markdown script to generate the experiment file, the html file to run the experiment, and a folder to save the data and document the experiment.

Browser-friendly experiments have several benefits. First, scripts are open source and easy to use and share (no installation is required to run an experiment). Scripts can be shared in a self-contained manner such that the experiment is reproducible and the methods are transparent and verifiable. Third, because the experiment is an HTML file it can be shared and run as part of the project website, facilitating understanding of the experimental procedures. Sharing experiment source code with a demonstration could benefit the review process and readers’ understanding of the procedures.

## Sharing and communicating

Above, we discussed using R Markdown, R package standards, and other R resources for creating research products. The next step is sharing and communicating these products. When the products are created as suggested above, *vertical* facilitates sharing the entire project as an R package for easy access to the data and functions, as a version controlled repository for the project’s source code, and as a website, where the components can be viewed in their final format.

### Sharing the source code with version control

The core of our proposal is that all project materials are stored in one directory on the user’s local filesystem. When version control, such as Git, is enabled in this directory, the entire project can be seamlessly shared with others through online platforms such as GitHub, GitLab, and OSF. Version control systems provide filesystems with additional functioning for keeping track of file versions, improved options for collaborating, and seamless integration with online collaboration platforms. For example, old versions of manuscripts are retained in the project’s history. However, the details are outside our scope, and we refer readers to Vuorre and Curley (2018). By default, *vertical* projects enable Git for new projects.

Because of the integration between Git(Hub) and one’s local filesystem, users should primarily interact with Git(Hub). However, for long-term preservation, users should also link the project’s GitHub repository to OSF. This can be done through the “Settings” page on an OSF project’s page. Then, one can create an OSF registration of that project, which preserves the GitHub repository’s current status on OSF permanently. This way, users gain both GitHub’s convenience and OSF’s long-term preservation.

### Sharing the project as a website

**Fig. 4:**
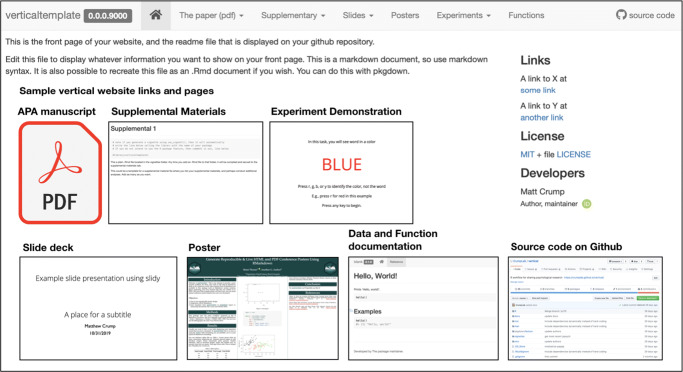
Research assets from a *vertical* project displayed on a website. The navigation tab bar on the top links to the content pages depicted in the body of the webpage

An additional benefit of the R package organizing principles is that the entire project can be easily showcased as a website (see Fig. 4). The website is compiled by *pkgdown* (Wickham & Hesselberth, 2020) (click “Addins” -> “Build Vertical” in RStudio), which is automatically included with *vertical* projects. For instructions on customizing the website, see https://pkgdown.r-lib.org/.

Building a *vertical* project as shown above creates the website in the docs folder, from where it can be viewed. During this process, all the R Markdown files within the project are evaluated, ensuring that all analyses, and thus products, are up to date and use the same data in the same computational environment. The website is easily made available on the internet by integrating the project with a GitHub repository (Vuorre & Curley, 2018), and then enabling GitHub pages under the repository’s settings. See https://crumplab.github.io/vertical/ for an example.

### Sharing as an R Package

If the project contains R functions, data, and documentation following the R package standard, then others can install the package on their computers for fast access to the data and functions from R. Again, integrating the project with GitHub is beneficial, because then others can install the package from GitHub using the *devtools* package, as was shown above in how to install *vertical*. We recommend sharing most data packages through GitHub (as discussed here), rather than the official CRAN channel. Sharing as an R package is very convenient for other R users, as any packaged data and functions are immediately available for use, natively in R, after package installation. We expand on the extended benefits of using R packages for transparency and reproducibility in the general discussion.

### Reproducibility

It is not uncommon to discover breaking changes in previously working code as a result of updates to a computational environment (e.g., a new language version or library dependency). As a result, an important component of computational reproducibility is specifying the (software) environment in which the computations were done, or should be done. In general, there is a continuum of static to dynamic approaches to this problem. A static approach could be a list of the requirements for the computational environment; whereas, a more dynamic approach could be a website or other container that actively runs the required environment (e.g., cloud computing tools such as Binder (https://mybinder.org/), CodeOcean (https://codeocean.com/), or RStudio Cloud (https://rstudio.cloud/)).

For simplicity, we adopt R packages as a robust static solution. For example, an R package’s metadata in the DESCRIPTION file can and should include the R packages and their versions that are required for the project. This provides a clear and standardized location for declaring the computational environment necessary to run the code in the project. Conveniently, end users can opt to install the package with dependencies, which automatically installs libraries listed in the DESCRIPTION file. However, even though the DESCRIPTION may describe the necessary computational environment, an end user may have to take additional steps to create it themselves (e.g., installing R on their computers). Nevertheless, this static approach is not incompatible with dynamic approaches, and advanced users could conceivably adapt vertical websites to link to cloud computing environments that preserve and execute project scripts.

## General discussion

We presented *vertical*, a stack of R tools for a single-platform solution for creating, curating, and communicating psychological research projects in a reproducible manner. It might seem that the benefits of this approach apply only to R users. We believe this is not the case; the workflow creates a single repository that includes all the research products related to a project, which can then be viewed as a public (or private, if desired) website and source code repository. Of course, to get the most out of the proposal, some R use is required. However, any standardized computational procedure assumes some computational environment, and our choice of R is natural due to its already widespread adoption in the psychological researcher community. Furthermore, by requiring raw data inclusion, our proposal does not strictly require using R. Also, the software we have discussed, RStudio, works well with other languages, including JavaScript, Python, and C++.

### Comparison to other approaches

We are not the first to recommend some form of standardization of workflows and procedures within psychology. First, Psych-DS is a collaborative project that aims to “promote the adoption of good practices in the management of scientific data” and “create a machine-readable format for these datasets that can support tools for analysis, discovery, and preparation of datasets in psychology” (https://github.com/psych-ds/psych-DS). Our suggestion is directly compatible with that effort, insofar as the R package standard doesn’t require any special format from the raw data. In fact, future work might bring these two approaches together to inform both how the raw data should be formatted (Psych-DS), and how research products based on that raw data could best be curated and shared (present work).

There are also at least two other R packages designed to help researchers organize their workflows and facilitate reproducibility and data/code sharing. We highlight these packages here, while also discussing their similarities and differences to our approach. *workflowr* is an R package that makes it easy to create version controlled and well-organized repositories of research materials, and share them as websites on GitHub (Blischak & Carbonetto, 2019); https://github.com/jdblischak/workflowr). The *workflowr* R package itself is quite mature, and provides helpful tools for compiling many R Markdown documents sequentially, but has a slightly different approach to our proposal, in that workflowr does not itself provide much of a standardized organization for the materials (although it does provide a template). Further, the materials are not organized as an R package, as they would be in the (extended) *vertical* approach. Additionally, *workflowr* does not easily incorporate other types of materials (manuscripts, slides, posters, experiments) in the resulting website, unlike *vertical*. However, it may be slightly easier to use otherwise, for the many useful functions that it provides.

Another approach is contained in the *rrtools* R package (Marwick et al., 2018); https://github.com/benmarwick/rrtools), which, unlike *workflowr* but like our approach, centers on the R package architecture. Additionally, *rrtools* helps set up a Docker container and continuous integration services for the project, both tools that we hope become more widely adopted in the future. However, it does not help users set up other important research materials as part of the project’s materials, such as slides and experiments, nor does it help users share their projects as websites.

More generally, however, tools and descriptions, such as Psych-DS, *rrtools*, *vertical*, and *workflowr* are pointing to the same important and general idea: Psychological sciences would benefit from standardizing, as much as possible, the ways in which computational work is curated. We think that these tools and principles are in their infancy, and their importance is only now being recognized. Therefore, we expect these tools to evolve and mutate to best suit the practical researchers’ needs, and hope that our suggestion provides some insight to how these tools might work in the future.

### Validating research assets

Our suggestion has wider implications for research transparency and reproducibility. Consider the implied truth claims associated with any peer-reviewed manuscript. The act of publishing can be seen as a validity assertion: that the truth claims made by the authors are verifiably true, or more weakly that the research assets are verifiable. Unlike a set of conclusions from premises that may be tested for logical validity, research conclusions and claims are not as easily scrutinized and tested. Reviewers trust that methods and results were as written when assets can not be verified directly. Sharing in the manner discussed here would facilitate scrutinizing and testing research projects, and thus potentially increase the reliability of the resulting scientific products.

Furthermore, organizing research assets in the manner discussed here allows computations to be formally tested, a process referred to as unit testing in software engineering. It is the practice of verifying that functions perform as intended in a variety of cases. We make a loose analogy between building a *vertical* project and unit testing. Building is a call to compile individual components of the project. If a user adheres to the *vertical* workflow, then R must be able to perform all of the scripted operations. Successfully building a *vertical* project is loosely analogous to performing unit tests that confirm and validate the process of asset creation. So, compiling a project is a test of the research project and verification its assets. Other researchers can perform the same verification, if the project’s source is shared. We note that *vertical* does not conduct proper unit tests on functions included in an R package. However, because these projects are R packages, proper unit tests can be easily included (see Wickham (2011)).

### Collaborating with vertical

Software developers routinely use version control platforms, such as GitHub and GitLab to coordinate software development across a team of collaborators (see, Vuorre and Curley (2018)). We recognize that scientific research is not software development. However, some components of the research workflow readily conform to the software development model—e.g., developing experiments and data analyses—and as such stand to benefit from borrowing best practices and tools from that domain.

Research projects can be hosted on GitHub throughout the lifespan of the project to make use its collaboration tools. With this in mind, *vertical* suggests using Git (along with its online platforms). For example, we used GitHub as a collaboration tool to create *vertical* and to write this manuscript (using *papaja*). Among other features, Git provides functionality similar to track changes in Microsoft Word that allow contributions to be reviewed, accepted, and modified. It makes this process robust even when many collaborators are involved. We also made extensive use of the GitHub issues tab, which is normally used to report bugs or feature requests to a software developer. Many users may find this feature very useful. In our case, we opened issues for discussion and then closed the issues once they were addressed. A side effect is that the GitHub issues tab, and the Git history, may double as a project development narrative, or a lab journaling system preserving issues and solutions for posterity.

### Teaching R with vertical

*vertical* may be most beneficial to users who already use R. Although our website tutorial is intended for R beginners, it is not a tutorial on the R language. Nevertheless, *vertical* could be used for pedagogical purposes, like a museum guide offering an orientation to collections in a large museum. For example, an R programming class for psychologists (or statistics/methods course using R) could be structured around the components of a vertical project, such as how to program functions, import and analyze data, write manuscripts, posters, slide decks, and websites in R. Moreover, *vertical* provides a convenient and well-organized workflow for students to save their work as they learn each component.

### Extending vertical

*vertical* is a modular open-source tool that can be extended by anyone. We included modules that we think are common, and can incorporate new modules as they are identified and suggested. Users can fork the *vertical* source code on GitHub, make suggested changes, and submit pull requests for review and inclusion.

Future work should also identify commonalities between our suggestion, and other related work such as rrtools (Marwick et al., 2018) and workflowr (Blischak & Carbonetto, 2019), and potentially work to unify these approaches to a more comprehensible and user-friendly tool for research project organization.

### FAIR

To maximize the reuse potential of open data and support “discovery through good data management”, Wilkinson et al., (2016) proposed the FAIR (Findable, Accessible, Interoperable, Reusable) guiding principles. Below, we describe how a *vertical* workflow targets FAIR principles and facilitates accessibility and data reanalyzes (FAIR principles paraphrased from, (Wilkinson et al., 2016; Martone et al., 2018)

**F** indable data are discoverable through persistent identifiers (e.g., permanent URLs / DOIs):

- Projects are accessible through the internet at OSF or GitHub
- DOIs for a *vertical* project can be minted through OSF or Zenodo

**A** ccessible data is available to approved researchers (e.g., anyone or lab members only) through a standardized communications protocol (e.g., Internet):

- Projects on GitHub and OSF can be made accessible to a specific set of users, or completely private, if desired
- Most data sharing guides emphasize manually uploading heterogeneous data files to a repository (e.g., OSF). R packages allow that, but also facilitate programmatic access to the data, and therefore are more accessible than methods relying on manual transfer of files

**I** nteroperable data is described (via metadata and file organization) in a common, openly available, non-proprietary language:

- R packages describe a strict organization of source files, resulting in a software product that is directly usable in a programming environment
- R packages describe a common language for documenting the data, its use, and dependencies
- R package (meta)data is human- and machine-readable and in a non-proprietary format
- Data wrapped in an R package is accessible in other programming environments due to standardized formatting

**R** eusable data are richly described and licensed to facilitate reuse:

- R packages are described in a standard format
- R packages facilitate use of appropriate licenses

## Conclusions

Borrowing standards and best practices from software development, such as the R package standard discussed here, may serve to dramatically improve reproducibility. By organizing research assets in a standard format, their reuse will be less time consuming and error-prone. Standard formats also facilitate large-scale meta-scientific investigations (Gorgolewski et al., 2016), and inclusion of data sets for meta-analyses. More generally, in addition to the convenience of a single-platform *vertical* workflow, adopting R standards during asset creation demands some amount of attention on how research products are curated, stored, documented, and shared. Drawing attention to these details may also serve to increase the reproducibility of our work.

## Footnotes

1 By “reproducibility”, we mean computational or analytic reproducibility by which previous results can be reproduced using existing data. Contrast this to “replicability”, in which entire procedures are repeated to generate new data to test a scientific idea.

2 https://crumplab.github.io/vertical

3 See also https://rmarkdown.rstudio.com/authoring_quick_tour.html for a quick overview of R Markdown.

4 https://crsh.github.io/papaja_man/introduction.html

5 https://bookdown.org/yihui/rmarkdown/slidy-presentation.html

6 https://crumplab.github.io/jsPsychr/
